# A Short Note about the Impact Action of a Water Jet Stabilized by a Coaxial Air Stream in the Air and Underwater

**DOI:** 10.3390/ma14175015

**Published:** 2021-09-02

**Authors:** Josef Poláček, Irena Marie Hlaváčová, Martin Tyč

**Affiliations:** Department of Physics, Faculty of Electrical Engineering and Computer Science, VSB-Technical University of Ostrava, 708 00 Ostrava, Czech Republic; josef.polacek@vsb.cz (J.P.); martin.tyc.st@vsb.cz (M.T.)

**Keywords:** pure water jet, coaxial air flow, jet stabilization, blasting, submerged jet operation

## Abstract

A new original method, applying a coaxial protective airflow, was tested aiming to improve the pure water jet efficiency in surface layer removal or medium hard materials cutting or blasting. The dual action of the air flow is expected: the air co-flowing the water jet with approximately the same velocity should prevent the central jet from breaking up into tiny droplets in the near field, and simultaneously, it should support jet decomposition into big parts with enough destructive potential in the far-field. A brief survey of the relevant literature dealing with the water jet instability is presented, introducing four recognized breakup regimes. An original cutting head designed to generate a waterjet surrounded by protective coaxial air flow is introduced. The submitted device is supposed to operate within the first wind-induced regime. Two types of experiments, consisting of blasting limestone bricks placed either in the air or underwater, were realized. The depths of kerfs produced with different water pressures and air overpressures were evaluated. While no substantial positive effect was recognized in the air performance, the submerged blasting of the same material under similar conditions appeared to be promising.

## 1. Introduction

The disintegration effect of a pure water jet impacting on the material is given by the impact pressure of the fluid droplets falling on the surface of the material. This pressure is proportional to the impact velocity of the droplets and the properties of the liquid (namely its density and speed of sound propagation). To maximize the impact action, it is desirable to achieve the decomposition of the water jet into regular drops [1,2]. Influencing the decay of the water jet by mechanical means, screens and the like, is problematic due to cavitation, which destroys the mechanical parts. Systems using ultrasonic water jet modulation [3] proved to be more reliable and thus have entered the industrial phase. Another way of pulsing jet generation, applying self-excited oscillations in the nozzle, has attracted waterjet researchers for years [4,5,6,7] but it still remains in the experimental phase although it should be more energy-saving and environmentally friendly. Quite recently, a brand new promising study introducing a self-excited pulse waterjet head applicable in mining for drilling holes and breaking rock was published [8]. The presented research is focused on the waterjet stability improvement in the near field and support of jet pulsation in the far-field which represents the operation zone of the device.

Stability and the decomposition process of a liquid stream are the main issues that must be solved to achieve sufficient effectiveness of the waterjet performance. The topic has been a widely studied problem, also thanks to a large number of other industrial applications. Knowledge of the laws of liquid jet behavior is important for spraying substances in the preparation of the coatings [9], for inkjet printers [10,11], diesel injection equipment [12], for fuel injection into rocket engines [13], to produce nonwovens, and other applications [14,15]. Due to the large number of possible applications, the parameters of the liquid jet (flow rate, nature of the flow—laminar, transient, turbulent, etc.) may differ substantially, the liquids with different properties (density, viscosity, surface tension) are used and so there is plenty of literature dealing with the problem.

The first description of the liquid jet instability caused by surface tension was proposed by Rayleigh [16]. His theory describes the time and space evolution of an inviscid liquid column moving in a stationary gas. Weber [17] included the effect of liquid viscosity in his theory. Ohnesorge [18] classified flow regimes for rounded-edge nozzles according to the different values of Reynolds and Weber numbers. The development of more efficient diesel engines inspired extensive research of atomization of diesel fuel represented by Bergwerk [19]. Later on, the application of high-speed photography enabled a more detailed investigation of instabilities in liquid jets [20], which, inter alia, brought observation, that water jets discharging into surrounding air moving at the same speed as the jet remained relatively stable. Wang et al. [21] studied the decay of a non-fully developed turbulent water jet with a coaxial turbulent high-velocity air stream. He reported a transition from a ligament to a membrane-mediated breakup around an effective Weber number *We* ≈ 13.

Nowadays, the research goals are most often aimed at the improvement of the liquid jet atomization [22,23]; injection of the liquid into a turbulent gaseous jet flow or twin fluid nozzles are used to fulfill this task [24]. However, the coaxial gas flow can, on the other hand, serve to stabilize the liquid jet moving through the quiescent air [25,26].

The direct experimental study of the flow inside the nozzle is impossible both due to high pressures and small dimensions. Therefore, either numerical simulations [27], or measurements of the consequences, e.g., interaction between the jet and solid medium-hard material, are performed. The aim of our work is to streamline the action of the water jet on the surface of the processed material by eliminating unwanted secondary decay of the water jet, which is caused by shear forces at the interface of the water jet and ambient air (either non-moving or moving with a speed different from the liquid jet velocity). We want to eliminate the undesirable aerodynamic effects by making the coaxial gas stream move at a similar speed as the water jet. This might reduce the undesired fragmentation of water droplets caused by the action of aerodynamic forces having arisen at the liquid-air interface due to the difference between the velocities of the two fluid streams.

The subsequent step of the research is the realization of experiments aiming to test the possibilities of application of the water jet with coaxial air flow for material erosion or cleaning below the water surface. The change of the environment substantially affects the structure of the jet in the target area and its interaction with materials. 

## 2. Theoretical Background

Let us first consider a cylindrical water jet flowing through a circular orifice into a quiescent gas environment. The fluid-dynamic instabilities inherent in the fluid flow result sooner or later in the disintegration of the continuous flow which is commonly referred to as jet breakup. Various breakup regimes should be distinguished; according to [28] there are four of them: Rayleigh regime, first wind-induced regime, second wind-induced regime, and atomization regime. It should be mentioned, however, that some researchers startup with a special, so-called “dripping” regime [27] when the flow is so slow that the breakup is driven mainly by gravity, producing relatively large droplets.

The nature of the liquid and gas flow is determined by the dimensionless Reynolds number

(1) $$Re=\frac{\rho vd}{\eta }=\frac{vd}{\nu },$$

where *ρ* (kg·m^−3^) is the density of the liquid, or gas, respectively, *v* (m·s^−1^) is the velocity of the liquid, or gas, respectively, *d* (m) is the diameter of the stream, *η* (Pa·s) is the dynamic viscosity and *ν* (m^2^·s^−1^) is the kinematic viscosity. The critical Reynolds number, for which a flow undergoes a transition from laminar to turbulent, is considered to be 2300 for fully developed pipe flow [29].

The type of interaction between flowing coaxial streams of liquid and gas, as far as the surface tension is concerned, can be characterized by the Weber number [30],

(2) $$We=\frac{\rho_{l}{\left(v_{l}-v_{g}\right)}^{2}D_{l}}{\sigma },$$

where *ρ_ℓ_* is the density of the liquid, *v_ℓ_* and *v_g_* are the velocities of the liquid and the air respectively, *D_ℓ_* is the diameter of the liquid jet and *σ* (N·m^−1^) the surface tension at the water-air interface; while the Ohnesorge number,

(3) $$Oh=\frac{\eta_{l}}{\sqrt{\rho_{l}D_{l}\sigma }},$$

where *ƞ_ℓ_* is the dynamic viscosity of the liquid, accounts for the effects of the viscosity.

At low velocities, corresponding to pressures not exceeding 20 MPa, which comprise the first two breakup regimes, the liquid breakup process is believed to initiate from long-wavelength small-amplitude disturbances raising at the liquid–gas interface. When the Reynolds number (Equation (1)) does not exceed 10^2^, the liquid tends to reach a state of minimum surface energy, a round jet of diameter *D_ℓ_* breaks into equal segments whose length is about 4.5 *D_ℓ_* [31]. The upper Reynolds number limit for this regime depends on the Ohnesorge number (Equation (3)); the liquid viscosity stabilizes the jet and increases the size of the created droplets. At larger Reynolds numbers, the jet becomes wavy because of aerodynamic effects; a regime called non-axisymmetric Rayleigh breakup, or first wind-induced regime, develops. The breakup sizes are of the order of the jet diameter in both these regimes, the drops are pinched off from the end of the jet [25]. The diameters of the drops are large enough to enable impact erosion on material caused by the water hammer effect.

The remaining two regimes produce drop sizes much less than the jet diameter, therefore the impact on the solid material is not so efficient. Another important difference between the two pairs of regimes is changes in the unbroken length (liquid intact length) *L_b_* denoting the distance from the nozzle exit where breakup begins, the liquid core length *L*, and the length needed for the liquid jet to be completely broken into drops and ligaments [32]. In the Rayleigh regime, the intact length increases with the Reynolds number until *L_b_*/*D_ℓ_* ≈ 10^2^, then decreases to about *L_b_*/*D_ℓ_* ≈ 10 before reaching a second maximum in the wind stress regime also called second wind-induced regime. Beyond this point, the intact length reduces to an asymptotic value *L_b_* ≈ 0 in the fully developed spray regime. The liquid core length *L* coincides with *L_b_* in the Rayleigh regimes but it is generally considerably longer otherwise, as the breakup of the jet is rising from the surface perturbations and the intact length approaches zero in these regimes.

In the presence of a high-speed coaxial air flow, which is the topic of this research, the breakup of the jet is substantially different as the exchange of momentum between the two fluids becomes the most important factor of the process. A lot of researchers have studied this problem, additional break regimes were observed. In [33], breakup lengths of round liquid jets in annular coaxial air streams were studied and a formula for the calculation of liquid intact length was published

(4) $$L_{b}/D_{l}=0.5We_{l}^{-0.4}Re_{g}^{0.6},$$

where *L_b_* (m) is the liquid intact length, *D_l_* (m) is the central tube inner radius, and the Weber and Reynolds numbers are based on the relative velocity between the gas and the liquid (Equations (2) and (5))

(5) $$Re_{g}=\frac{\rho_{g}\left(v_{g}-v_{l}\right)\left(D_{g}-D_{l}\right)}{\eta }\;,$$

The above-mentioned theories provide so-called linear stability analysis [30]. They suppose that unstable disturbances are convected downstream in a group with a certain group velocity and gradually amplify in time to such an extent that breakup of the jet occurs. However, the whole process is basically highly non-linear, therefore, any theoretical description is always based on considerable simplification and, generally, the exact agreement with the experiment cannot be expected.

## 3. Materials and Methods

**Figure 1:**
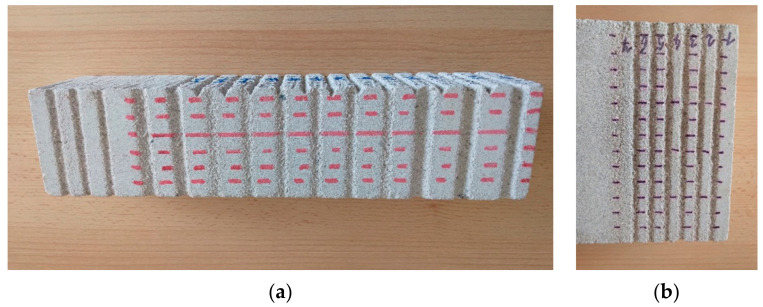
(**a**) an example of cutting in the air; (**b**) an example of submerged cutting.

The lime-sand bricks (KM Beta, Hodonín, Czech Republic), with a compressive strength of 30 MPa were used in the experiments, as a model of the material that should be removed from the remediated surfaces in practice. The bricks were blasted transversely with a traverse speed of 100 mm per minute (Figure 1). The compressive strength of the bricks was measured by the MTS 816 Rock Test System (MTS Systems Corp., Eden Prairie, MN, USA). The original dimensions of the brick were 290 × 140 × 65 mm; due to a shortage of material, some bricks were used twice in our experiments—they were cut lengthwise, and the resulting cut surface was blasted as well (Figure 1a, the vertical wall). The whole cut (140 mm) was measured with a digital caliper PWA (Bernardo, Linz, Austria); the depth was measured for each cm of the grove (the marks on measured points are visible in Figure 1b) and the depths for each kerf were averaged to obtain the mean value. The standard deviation of the measured values was evaluated for each kerf as well. The caliper is able to register deviations of 0.01 mm.

The dynamic viscosity of water is 1.002 × 10^−3^ Pa·s at room temperature and normal pressure (20 °C; 101,325 Pa) [34] and the dynamic viscosity of air is 18.25 × 10^−6^ Pa·s under the same conditions [35]. With a water nozzle diameter of 1.0 mm and a water speed ranging from 81 to 119 m·s^−1^, Reynolds’ number exceeds 8 × 10^4^, therefore, the investigated water flow is turbulent. The inlet air flow diameters are 4 mm, the outflow has an annulus shape, therefore, the characteristic length should be the difference between the inner and outer diameters, i.e., 1.8 mm. The Reynolds number for the air flow with velocities equal to the speeds of the water jet reaches values around 30,000, therefore, the flow should also be clearly turbulent.

The water jet was generated by the pump Kränzle 3270 (Kränzle, Illertissen, Germany) at constant pressures of 10 MPa, 15 MPa, or 20 MPa, the last one corresponds to the speed of the water jet 119 ms^−1^ (measured from the volume flow—the amount of liquid trapped in the collection vessel at a given working pressure of the pump). The water pressure was measured in the pump; the air pressure was measured on one feeding pipe by means of a pressure gauge; it varied from 0 to 0.25 MPa; the zero pressure represented cutting without air assistance.

We performed two different types of experiments both with a special cutting head prepared according to the original design of one of the authors (J.P.). First, the measurement in the air was realized representing the introductory tests. The cutting head fitted with a brass water nozzle was equipped with two side openings and a small relaxation chamber to enable the formation of the coaxial air flow around the nozzle outlet. The head was placed in the air, as well as the sample of the examined material. The goal was to study the way of interaction between a highly turbulent water jet and a coaxial air flow. We tried to search for the conditions leading to stabilization of the water jet (positively affection) and on the contrary to identify conditions accelerating the atomization of the water jet and, therefore, causing reduction of the efficiency. The elucidation of these conditions is important for the determination of suitable parameters when applying a water jet in conjunction with a coaxial air stream, especially for submerged applications.

We investigated the disintegration effect on the surface of a material caused by a pure water jet and a water jet cooperating with a coaxial air stream. The situation when the air flow has the same velocity as the water jet is considered to be optimal as under such conditions the relative Weber number (Equation (2)) is zero and the surface interaction between the two fluids is minimized. Measuring the speed of the water jet and the air flow all at once is experimentally very demanding, therefore, we applied the following procedure instead: The air was supplied from the tank through a pipe with a throttle valve, which allowed regulation of the amount of air and thus the speed of its flow. The optimal speed of the air flow can be found by looking for a setting at which there is minimal entrainment of water drops from the surface of the water jet. We tried to assess this condition visually using the water pressure 20 MPa and tuning the air overpressure, but the observation was not convincing enough. We were neither able to establish such a condition exactly, nor measure the velocity of the air flow. Therefore, the evaluation of the results of cutting experiments remained the only reliable information. Based on this key the cutting-blasting experiments with different water pressures and air overpressures were performed in the air with a stand-off distance of 180 mm.

**Figure 2:**
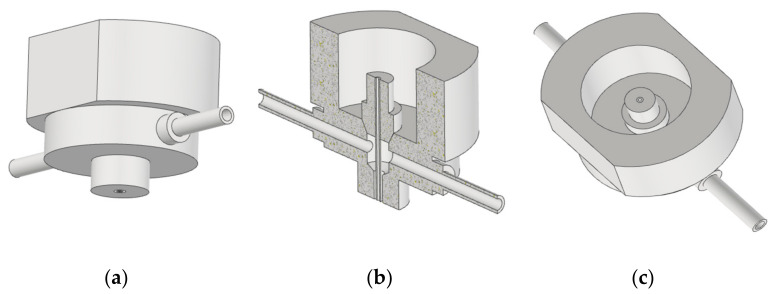
The bottom part of the combined air/water cutting head: (**a**) overall view; (**b**) vertical cut; (**c**) inside view.

**Figure 3:**
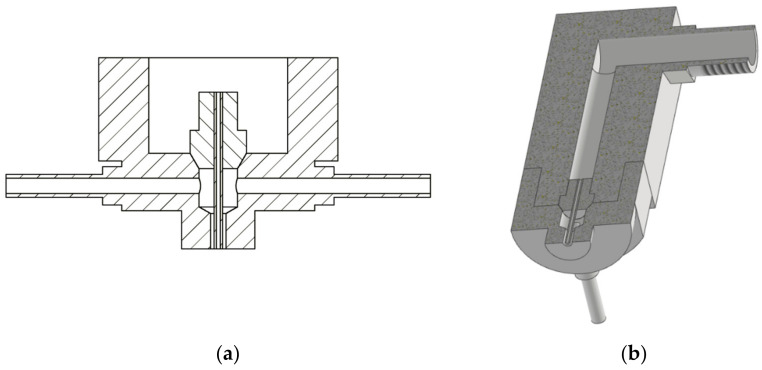
Scheme of the cutting head equipped with an air supply: (**a**) vertical cut through the bottom part; (**b**) overall cut along the whole cutting head, one of the air feeding pipes can be seen below.

The second type of experiment was measurement below the water surface. Both the head with a nozzle for water and coaxial air flow and the examined material sample were submerged. Two stand-off distances, 60 mm and 120 mm, were applied. These experiments were realized aiming to study the possibility of jet impact intensification, in case of submerged material cutting, by means of coaxial air flow. In order to improve the jet flow coherence, a steel needle with an inner diameter of 1 mm was inserted into the original nozzle. The air was delivered through two cylindrical pipes with a diameter of 4 mm placed symmetrically opposite to each other into the central air cavity and then proceeded through an air pipe with a diameter of 4 mm along the outer needle wall (Figure 2 and Figure 3). 

The air feeding extension is made of duralumin and is firmly screwed on the main body of the head (Figure 3b). The main body of the cutting head was made of steel.

To compare the experimental results with available theories it was necessary to calculate the velocity of the air flow. We can suppose that the absolute air pressure in the feeding piping is approximately the same as the pressure in the relaxation chamber and it is equal to normal pressure plus overpressure measured on the feeding piping. Denoting *v_1_* the velocity of the pressurized air in the feeding pipe, *v_2_* the velocity of the air at the nozzle outlet, and supposing there are no leaks anywhere, we can write the continuity equation in the form

(6) $$A_{1}\rho_{1}v_{1}=A_{2}\rho_{2}v_{2},$$

where *A*_1_ and *A*_2_ (m^2^) are cross-sections of the air inlet and outlet, respectively, and *ρ*_1_ and *ρ*_2_ (kg·m^−3^) are densities of the air under overpressure and under normal pressure. We can derive the relation between air density and pressure from the ideal gas law:

(7) $$pV=nRT=\frac{m}{M}RT\Rightarrow \varrho =\frac{p}{rT},$$

where *p* (Pa) is absolute pressure, *V* (m^3^) is volume of the gas, *n* (mol) is the amount of substance, *m* (kg) is mass, *M* (kg·mol^−1^) is molar mass, *R* = 8.314 J·mol^−1^·K^−1^ is universal gas constant, *T* (K) is absolute temperature and *r* (J·kg^−1^) is specific gas constant. Finally, the Bernoulli equation provides relation from which the air velocity can be calculated when Equations (6) and (7) are substituted into it

(8) $$p_{1}+\frac{1}{2}\rho_{1}v_{1}^{2}=p_{2}+\frac{1}{2}\rho_{2}v_{2}^{2}\Rightarrow p_{1}+\frac{1}{2}\frac{p_{1}}{rT}{\left(\frac{A_{2}p_{2}}{A_{1}p_{1}}\right)}^{2}v_{2}^{2}=p_{2}+\frac{1}{2}\frac{p_{2}}{rT}v_{2}^{2}$$

(9) $$v_{2}^{2}=\frac{2rT\left(p_{1}-p_{2}\right)}{p_{2}\left[1-\frac{p_{2}}{p_{1}}{\left(\frac{A_{2}}{A_{1}}\right)}^{2}\right]}\;$$

The numerical parameters necessary for calculation are *M* = 28.964 × 10^−3^ kg·mol^−1^, *r* = 287 J·kg^−1^, *T* = 293.15 K (*t* = 20 °C), *p*_2_ = 101,235 Pa (normal pressure), *p*_1_ = *p*_2_ + Δ*p*, *A*_1_ = 2.51 × 10^−5^ m^2^ (two cylindrical pipes with diameters 4 × 10^−3^ m), *A*_2_ = 8.77 × 10^−6^ m^2^ (anulus with an inner diameter 2.2 × 10^−3^ m and an outer diameter 4 × 10^−3^ m).

## 4. Results and Discussion

The first series of experiments were test cuts for different distances from the sample surface (180 mm, 120 mm, 80 mm). The cuts were performed using water pressure 20 MPa and air overpressure 0.01 MPa. The kerf depths without and with airflow assistance under otherwise identical conditions (distance from the cut material surface) were compared. The results are presented in Table 1. This measurement was aimed at proper choice for optimum standoff distance. On the basis of this measurement, a standoff distance of 180 mm was chosen as the optimum standoff distance leading to sufficient breakup of the jet and intensification of the impact on solid material. This choice may retrospectively be questioned in comparison to percentage improvement (third column) but the absolute value of the kerf depths was also taken into account when making the decision.

In the bottom right corner of the table, there is supplementary information about the cutting—namely velocities of both fluids in the outlet of the cutting head. The air velocity, however, is strongly overestimated, it is a theoretical value neglecting all the losses in the piping calculated according to the formula derived in Equation (9). Therefore, the comparison of the experimental data with Equation (4) did not bring an agreement, the theoretical value was much smaller than measured values. The reason might be either an overestimation of the velocity of air or non-applicability of the theory for the studied experiment (the breakup regime might be different).

The results of the initial measurement proved that co-flowing air improves the erosivity of the medium pressure waterjet. A series of detailed measurements were realized with three different water pressures and five air overpressures.

The evaluated parts of the test kerfs were about 12 cm long (the start and end parts of the kerf were not included in the evaluation in order to avoid undesirable marginal phenomena because of some dependence on the vicinity of the edge of the block of the test material was observed), their depth was measured at distances of 1 cm (i.e., ten points per kerf). The results were processed statistically. The results of the kerf depth measurement were oscillating around the mean value with a fairly large uncertainty (Table 2); however, the variance of the results rather gives information about the degree of inhomogeneity of the used test material. It is obvious that the best impact was produced with the least value of air pressure (0.05 MPa). Higher air pressures do not improve the impact effect sufficiently; on the contrary, they rather amplify the jet instabilities and support jet atomization. The coaxial air flow with pressure 0.25 MPa deteriorates the jet performance for all measured pressures of water.

**Figure 4:**
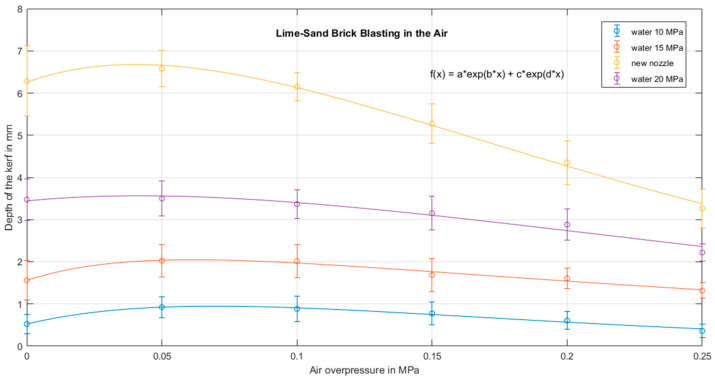
Influence of coaxial air flow on average kerf depths for water pressure 10–20 MPa.

The graph in Figure 4 shows the dependence of the average kerf depth on the air pressure at the nozzle inlet when measured in air. The positive stabilizing effect of the coaxial air flow at lower air pressures (up to 0.1 MPa) was not as distinct as we expected, the worst performance was observed with the highest water pressure, 20 MPa. The positive influence of air flow seems to be almost negligible; the fit curve is very flat in this case. This result corresponds with the initial measurement, and it might indicate that the standoff distance should be shorter.

Another important finding is that at the air overpressures below 0.1 MPa there is no significant disintegration of the water jet due to interaction with the air. At higher air inlet pressures (above 0.1 MPa), which correspond to a higher air flow velocity, the effect of the airflow seems to act opposite to what was intended, water jet probably disintegrates into small droplets due to the highly turbulent co-flowing air. As a result, the impact action of the water jet is smaller, the kerf is shallow and wide. The polynomial approximation proved to be insufficient, the maximum position of the polynomial trendlines did not match the experimental data. Therefore, the exponential fit was applied using the MATLAB program (R2020b academic use, 2020, MathWorks, Natick, MA, USA). The details indicate that the kerf depth reaches its maximum around the air pressure of 0.05 MPa. The exponential fit seems to indicate that the local maximum should occur at approximately 0.06 MPa. This experimental finding might be in accordance with the theoretical analysis if the energy losses of the air flow are more than 50%. However, another even more important question is the relative erosivity improvement achieved by the proposed technology. The experimental results seem to indicate that application of the protective coaxial air flow for pure jet operation in the air is not promising, the improvement of the erosivity is too small.

Retrospective evaluation of the experiments in the air, namely comparison of depths of kerfs produced in different stand-off distances, led us to reevaluate the optimal standoff distance—120 mm appears to be a better choice for a smooth cylindrical nozzle.

**Figure 5:**
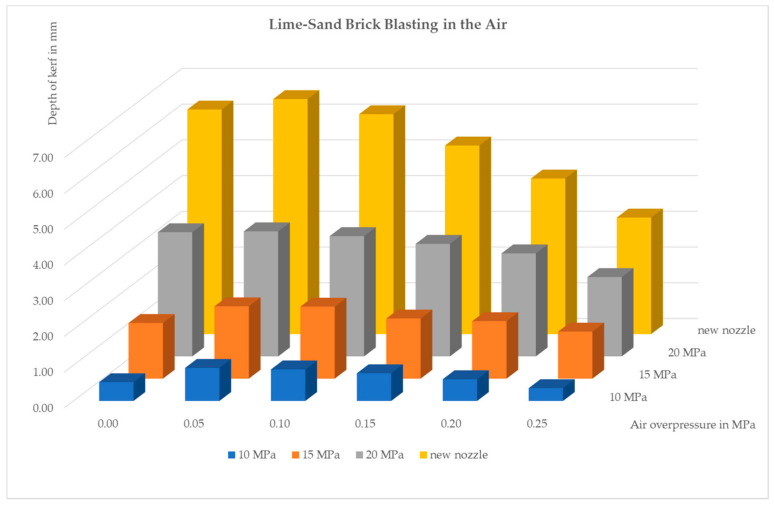
The average kerf depths after blasting with and without protective coaxial air flow with water pressures 10, 15, 20 MPa; the first column on the left represents blasting without air flow.

Another substantial improvement in the jet operation is possible when a modified nozzle shape with conical input is applied. The results acquired with such nozzle and water pressure 20 MPa are presented in Figure 4 as a “new nozzle” graph. A bar graph is also provided (Figure 5) to make the results more illustrative.

**Figure 6:**
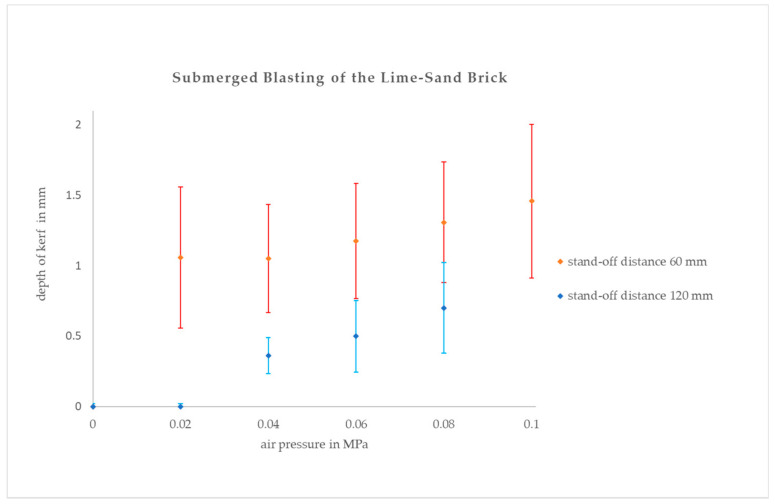
Influence of coaxial air flow on average kerf depths for submerged blasting with water pressure 20 MPa and two different stand-off distances.

The graph in Figure 6 shows the air overpressure dependence of the average kerf depth during submerged blasting. Based on the evaluation of the in-air experiments and theoretical analysis, we used lower air inlet pressures, which, as we assumed, do not lead to destabilization of the water jet. The results indicate that without a protective coaxial air stream, the water jet is quickly attenuated and it has almost no effect on the investigated material (Figure 1b, the kerf №. 7). When using a coaxial air stream, the water jet exhibits a destructive effect on the material in both stand-off distances which were tested, and this effect increases with increasing air inlet pressure up to 0.1 MPa.

The results of the submerged operation of the proposed device are, therefore, much more promising, but they are rather surprising in the meantime. We were not able to determine exactly the velocity of the air flow, it was obvious that it should substantially differ from the theoretical value calculated from the measured value of overpressure at the inlet. Therefore, we had to estimate the real value of this velocity according to the kerf depths measurement. The results of the in-air experiments indicated that the energy losses should have been more than 50%. However, the submerged experiments seem not to prove such an assessment; the energy of the airflow was big enough to sufficiently protect the jet in the water environment as far as 120 mm from the nozzle outlet supposing the air overpressure exceeded 0.04 MPa. It is obvious that the character of the submerged air flow is different, and it would be necessary to study it in a more detailed way. Numerical simulation similar to [36,37] might be helpful, although recent research works are focused primarily on the jet breakup improvement which is not the goal that we are aiming for.

The trendlines for the submerged experiments are quite different from the in-air ones. They are more linear and both of them are growing monotonously in the range of applied overpressures. It can be expected that the jet has limited outreach even with air support but due to the technical limitation of our cutting device, it was not possible to realize the submerged measurement with a bigger stand-off distance.

Although the idea of utilization of coaxial air flow for improvement of the waterjet performance has already appeared in the last decade; it was studied related to abrasive waterjets, either injection [27] or suspension [38] ones. Our research, therefore, represents a brand new idea that should widen the range of waterjet applications.

## 5. Conclusions

A new method of improving the performance of a pure medium pressure water jet was introduced and tested. Waterjet protected by a co-flow of air generated coaxially in a specially designed cutting head was applied for limestone bricks cutting either in the air or underwater. The research work brought the following important findings:

- The positive stabilizing effect of the coaxial air flow was observed in the in-air experiments at air overpressures not exceeding 0.1 MPa.
- The airflow generated by overpressure below 0.1 MPa does not give rise to jet breakup in the near region; the positive protective influence prevails.
- The most effective relative improvement of erosivity, 17 percent, was observed with a water pressure of 20 MPa, an air overpressure of 0.01 MPa, and a standoff distance of 12 cm.
- At air inlet pressures above 0.1 MPa the effect of the airflow acted opposite to what was intended—water jet disintegrated into small droplets due to the highly turbulent co-flowing air.
- The effectivity of the new cutting head performance in the air can be to a great extent improved by the application of a water nozzle with the conical inlet.
- The results of the submerged experiments indicate that without a protective coaxial air stream, the water jet is quickly attenuated and has almost no effect on the investigated material. When a coaxial air stream is applied, the water jet exhibits a destructive effect on the material in 60 mm as well as in 120 mm, if proper air overpressure is applied.

The results of the experiments showed that the water jet in conjunction with the coaxial air stream provides promising results when used below the water surface. As both water and air working pressures were intentionally relatively low, our device appears to be more economically advantageous and cost-effective. The area of application is aimed at processes in which removal of surface, impaired, or less adherent layers of material is demanded, i.e., cleaning of surfaces below the water surface remediation of damaged surfaces or rock drilling in the oil industry.

## Figures and Tables

**Table 1:** Average depth of kerfs produced by air blasting in three different stand-off distances.

Stand-Off Distance	Air Overpressure (MPa)		Increment	Additional Information
	0	0.01		
80 mm	1.19 ± 0.20.	1.26 ± 0.15	6%	Water pressure 20 MPa ⇒ *v_ℓ_* = 119 m·s^−1^ Air overpressure 0.01 MPa ⇒ *v_g_* = 133 m·s^−1^
120 mm	1.96 ± 0.29	2.28 ± 0.19	17%	
180 mm	3.25 ± 0.41	3.30 ± 0.31	2%	

**Table 2:** Average depth of kerfs produced during the air blasting.

Air Overpressure in MPa	0	0.05	0.10	0.15	0.20	0.25
Water pressure 10 MPa	0.53 ± 0.22	0.93 ± 0.25	0.88 ± 0.31	0.78 ± 0.27	0.61 ± 0.21	0.36 ± 0.16
Water pressure 15 MPa	1.56 ± 0.46	2.03 ± 0.39	2.02 ± 0.40	1.68 ± 0.39	1.61 ± 0.24	1.32 ± 0.18
Water pressure 20 MPa	3.48 ± 0.49	3.50 ± 0.42	3.37 ± 0.34	3.15 ± 0.40	2.88 ± 0.37	2.22 ± 0.21
New nozzle with cone inlet	6.28 ± 0.83	6.58 ± 0.43	6.15 ± 0.34	5.28 ± 0.47	4.35 ± 0.52	3.26 ± 0.46

## Data Availability

The data presented in this study are available on request from the corresponding author.
